# MTBVAC-Based TB-HIV Vaccine Is Safe, Elicits HIV-T Cell Responses, and Protects against *Mycobacterium tuberculosis* in Mice

**DOI:** 10.1016/j.omtm.2019.01.014

**Published:** 2019-02-07

**Authors:** Esther Broset, Narcís Saubi, Núria Guitart, Nacho Aguilo, Santiago Uranga, Athina Kilpeläinen, Yoshiki Eto, Tomáš Hanke, Jesús Gonzalo-Asensio, Carlos Martín, Joan Joseph-Munné

**Affiliations:** 1 Grupo de Genética de Micobacterias, Departamento de Microbiología y Medicina Preventiva, Facultad de Medicina, Universidad de Zaragoza, C/Domingo Miral s/n, Zaragoza 50009, Spain; 2 CIBER Enfermedades Respiratorias, Instituto de Salud Carlos III, Madrid, Spain; 3 Instituto de Biocomputación y Física de Sistemas Complejos (BIFI), Zaragoza, Spain; 4 AIDS Research Group, Hospital Clínic de Barcelona/IDIBAPS-HIVACAT, School of Medicine, University of Barcelona, Barcelona, Catalonia, Spain; 5 Red Temática de Investigación Cooperativa en SIDA (RD12/0017/0001), Spanish AIDS Network, Madrid, Spain; 6 EAVI2020 European AIDS Vaccine Initiative H2020 Research Programme, London, UK; 7 The Jenner Institute, Nuffield Department of Medicine, University of Oxford, Oxford, UK; 8 Servicio de Microbiología, Hospital Universitario Miguel Servet, IIS Aragón, Zaragoza, Spain; 9 Servei de Malalties Infeccioses, Hospital Clínic de Barcelona, Catalonia, Spain

**Keywords:** HIV, tuberculosis, vaccine, MTBVAC, BCG, mycobacterium, HIVA, p2auxo, SCID, BALB/c

## Abstract

The tuberculosis (TB) vaccine MTBVAC is the only live-attenuated *Mycobacterium tuberculosis* (*Mtb*)-based vaccine in clinical development, and it confers superior protection in different animal models compared to the current vaccine, BCG (*Mycobacterium bovis* bacillus Calmette-Guérin). With the aim of using MTBVAC as a vector for a dual TB-HIV vaccine, we constructed the recombinant MTBVAC.HIVA^2auxo^ strain. First, we generated a lysine auxotroph of MTBVAC (MTBVACΔ*lys*) by deleting the *lysA* gene. Then the auxotrophic MTBVACΔ*lys* was transformed with the *E. coli*-mycobacterial vector p2auxo.HIVA, harboring the *lysA*-complementing gene and the HIV-1 clade A immunogen HIVA. This TB-HIV vaccine conferred similar efficacy to the parental strain MTBVAC against *Mtb* challenge in mice. MTBVAC.HIVA^2auxo^ was safer than BCG and MTBVAC in severe combined immunodeficiency (SCID) mice, and it was shown to be maintained up to 42 bacterial generations *in vitro* and up to 100 days after inoculation *in vivo*. The MTBVAC.HIVA^2auxo^ vaccine, boosted with modified vaccinia virus Ankara (MVA).HIVA, induced HIV-1 and *Mtb*-specific interferon-γ-producing T cell responses and polyfunctional HIV-1-specific CD8+ T cells producing interferon-γ (IFN-γ), tumor necrosis factor alpha (TNF-α), and CD107a in BALB/c mice. Here we describe new tools to develop combined vaccines against TB and HIV with the potential of expansion for other infectious diseases.

## Introduction

Today, tuberculosis (TB) has reached alarming proportions. An estimated 10 million people have developed TB in 2017 and 9% were people living with HIV (72% in Africa). There were an estimated 1.3 million TB deaths among HIV-negative people and an additional 300,000 deaths among HIV-positive people, as reported by the World Health Organization (WHO)^1^ in 2018. TB is poverty related with a major burden in the poor and developing parts of the world, and it is aggravated by the HIV-AIDS pandemic, which greatly increases the risk of the infection evolving into active TB disease.

HIV-AIDS is a major global public health issue. Between 2010 and 2016, new HIV infections fell by 11% in adults and 47% in children, and AIDS-related deaths fell by 48% since the peak in 2005. This achievement was the result of great efforts by national HIV programs supported by civil society and a range of development partners.^2^ However, sub-Saharan Africa accounted for 64% of new HIV infections in 2016, and, even though it is encouraging that 1.6 million people are currently receiving treatment in resource-poor settings, ensuring universal access to antiretroviral therapy still represents an enormous challenge.^3^ Thus, the development of effective, safe, and affordable vaccines against both diseases could have a tremendous impact on public health.

The risk of active TB is estimated to be between 16 and 27 times greater in people living with HIV than among those without HIV infection.^4^ *Mycobacterium bovis* bacillus Calmette-Guérin (BCG) has been the only licensed vaccine against TB for more than 90 years,^5^ but the BCG-induced protective effects against pulmonary disease over all ages are variable.^6^, ^7^ Nevertheless, BCG^8^ vaccination has several beneficial effects: (1) BCG vaccination reduces rates of *Mycobacterium tuberculosis* (*Mtb*) infection, aiding in the decrease of the pool of latent infections from which future cases of active disease may arise;^9^ (2) BCG provides strong protection against disseminated forms of the disease in infants and young children;^10^, ^11^ (3) BCG revaccination of adolescents may provide additional benefits for the prevention of TB;^12^ and (4) BCG vaccination reduces all-cause mortality through beneficial non-specific (heterologous) effects on the immune system.^13^, ^14^ These four effects strengthen the motivation for the inclusion of BCG in the global vaccination program.^15^

We previously constructed and characterized MTBVAC, the first and only live-attenuated *Mtb*-based vaccine candidate in clinical development against TB disease in the pipeline. MTBVAC contains two independent deletions in the *phoP* and *fadD26* genes without antibiotic resistance markers, and it fulfills the Geneva consensus requirements for progressing into clinical trials.^16^ The vaccine candidate MTBVAC was safe, and it conferred superior protection in different animal models compared to the licensed BCG reference strain in use today. To date, phase I trials in adults and neonates (ClinicalTrials.gov: NCT02013245 and NCT02729571) have been successfully completed, and phase II trials for dose definition, at birth and in adults with and without latent TB, are in progress (ClinicalTrials.gov: NCT02933281 and NCT03536117). Clinical results showed that MTBVAC was immunogenic, in a dose-dependent manner, and it had a similar safety profile as that of BCG.^16^, ^17^

It is well known that there is strong evidence in favor of a role for HIV-1-specific T cell responses in the control of HIV-1 replication.^18^, ^19^ One promising approach for T cell induction is *M. bovis* BCG as a bacterial live recombinant vaccine vehicle. Specific humoral and cellular immune responses against HIV-1 have been detected after immunization of mice with recombinant BCG (rBCG) expressing HIV-1 antigens.^20^, ^21^, ^22^, ^23^, ^24^ We previously developed several rBCG HIV-1 vaccine candidates with the aim of inducing protective cell-mediated responses. Confirming the efficacy of an HIV-1 vaccine candidate in humans is as of yet not possible in animal studies alone. Achieving protection against HIV infection in humans following active vaccination, and subsequently identifying the correlates of protection, would allow the validation of protection in animal models. Our aim is to induce a strong CD8+ T cell response capable of aiding and complementing the protective efficacy of antibody-based vaccines, while providing viral control in the case of an infection occurring.

Our starting platform was based on a heterologous rBCG prime and recombinant modified vaccinia virus Ankara (MVA) boost regimen delivering a common immunogen called HIVA (MVA.HIVA), which is derived from consensus Gag protein of HIV-1 clade A, prevalent in central and eastern Africa, and a string of human CD8^+^ T cell epitopes.^25^ Recently, we engineered a new BCG.HIVA^2auxo^ vaccine strain harboring an antibiotic-free plasmid selection system and maintenance. The BCG.HIVA^2auxo^ vaccine in combination with MVA.HIVA was safe, and it induced HIV-1 and *Mtb*-specific interferon-γ-producing T cell responses in adult BALB/c mice.^26^

In this study we have constructed a novel live-attenuated vaccine for HIV-1 and TB that is vectored by a lysine auxotroph of MTBVAC, MTBVAC.HIVA^2auxo^. This is an innovative approach to develop bivalent TB and HIV vaccines that could be administered at birth and with the potential to confer protection against both diseases.

## Results

### Construction and Characterization of a Lysine Auxotroph of MTBVAC, the MTBVAC*Δlys* Strain

**Figure 1:**
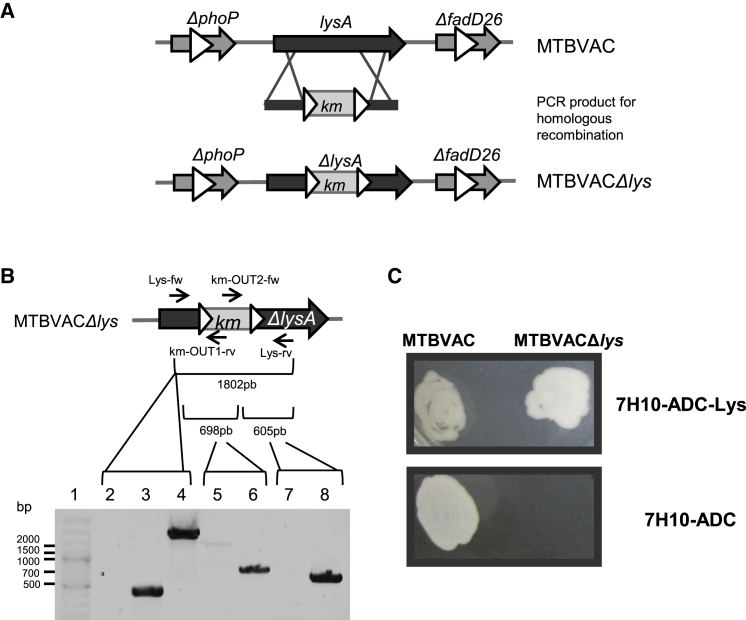
Construction and Characterization of MTBVACΔ*lys* Strain (A) *lysA* gene (gray arrow) from MTBVAC was inactivated using homologous recombination techniques by introducing a kanamycin resistance cassette (gray rectangle), flanked by two resolvase sites (white arrowheads) in order to allow the release of the resistance cassette. The inactivated *phoP* and *fadD2*6 genes in the MTBVAC parental strain are also illustrated. (B) Genotypic characterization of the *lysA* gene inactivation by the kanamycin cassette (km) insertion, primers used, and expected sizes of the PCR products are indicated. MTBVAC sample was used in lanes 3, 5, and 7 and MTBVACΔ*lys* samples in lanes 4, 6, and 8. Lane 1, molecular weight marker; lane 2, negative control; lanes 3 and 4, PCR product of the *lysA* gene using Lys-fw and Lys-rv primers; lanes 5 and 6, PCR of the 5′ insertion point of km expression cassette using Lys-fw and km-OUT-rv primers; and lanes 7 and 8, PCR of the 3′ insertion point of km expression cassette using km-OUT-fw and Lys-rv primers. Genes are represented as gray arrows; gray rectangles illustrate antibiotic resistance markers and white arrowheads depict resolvase recognition sequences or *res* sites. (C) Phenotypic lysine auxotrophic verification by plating MTBVACΔ*lys* strain in 7H10-ADC with and without lysine supplementation.

For MTBVAC*Δlys* construction, the previously described recombineering-based technique was used.^27^ *Rv1293* (*lysA*) gene, which codes for the last enzyme involved in Lysine (Lys) synthesis,^28^ was inactivated by homologous recombination with a PCR product containing a kanamycin (*Km*) resistance cassette disturbing the *lysA* gene (Figure 1A). To ensure proper selection of recombinants, the final MTBVAC transformed with the homologous PCR product *lysA-Km* was plated on 7H10 complete medium supplemented with Km and Lys. Correct recombination was confirmed by PCR using three different pairs of primers, which amplify the complete recombined region (Lys-fw/Lys-rv), the upstream (Lys-fw/km-OUT1-rv), or the downstream (km-OUT2-fw/Lys-rv) region (Figure 1B). After MTBVAC*Δlys* construction, Lys auxotrophy was confirmed by plating on 7H10-ADC with and without Lys supplementation (Figure 1C) and also by colony-forming unit (CFU) enumeration after removing Lys from medium (data not shown). Results showed the absence of MTBVAC*Δlys* growth in non-lysine-supplemented plates, whereas the MTBVAC strain grew at a similar level in both supplemented and non-supplemented plates. Accordingly, this auxotrophic MTBVAC*Δlys* strain was used in the subsequent experiments to generate recombinant vaccines expressing the HIVA immunogen.

### Construction and Characterization of the MTBVAC.HIVA^2auxo^ Vaccine Strain

**Figure 2:**
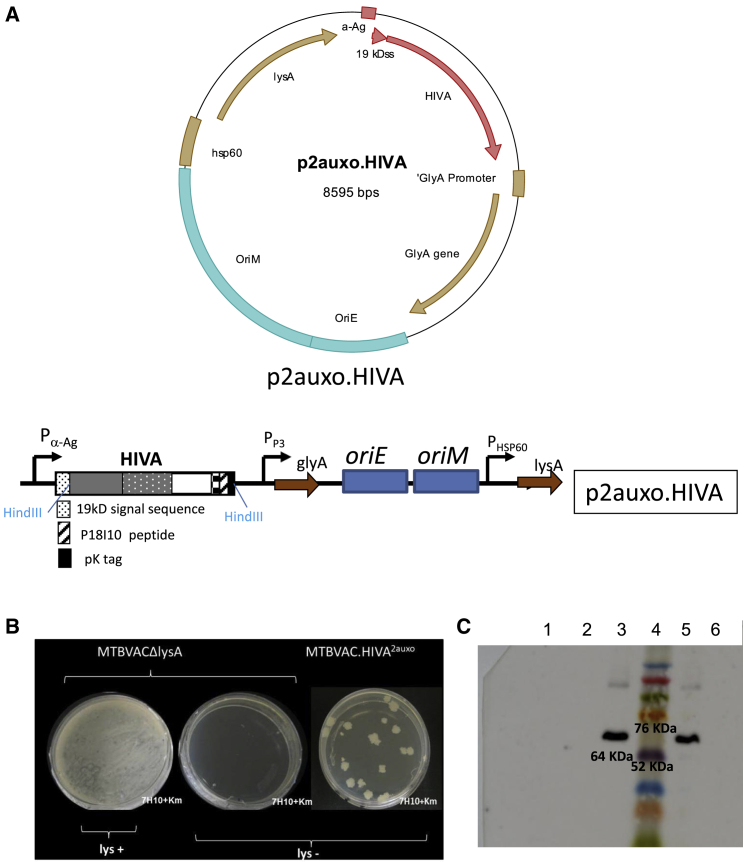
Construction of MTBVAC.HIVA^2auxo^ (A) The HIVA gene was fused to the region encoding the 19-kDa lipoprotein signal sequence of the episomal p2auxo.Ø *E. coli*-mycobacterial shuttle plasmid to obtain p2auxo.HIVA plasmid. The BALB/c mouse T cell and Mab-Pk epitopes used in this study are depicted. P α-Ag (*M. tuberculosis* α-antigen promoter), PHSP60 (heat shock protein 60 gene promoter), and *glyA*- and *lysA*-complementing genes are used as markers for selection and maintenance in *E. coli* M15Δ*Gly* and MTBVACΔ*lys*, respectively. (B) Phenotypic characterization of the lysine auxotrophy and plasmid complementation of MTBVACΔ*lys* and MTBVAC.HIVA^2auxo^ (MTBVACΔ*lys* plated on lysine-supplemented 7H10, left; MTBVACΔ*lys* plated on non-lysine-supplemented 7H10, center; and MTBVAC.HIVA^2auxo^ plated on non-lysine-supplemented 7H10, right). (C) Western blot of MTBVACHIVA^2auxo^ lysates. Lanes 1 and 2, MTBVAC.Ø^2auxo^ clones 1 and 2; lanes 3 and 5, MTBVAC.HIVA^2auxo^; lane 6, BCG wild-type lysate (negative control). HIVA immunogen was detected using the anti-Pk monoclonal antibodies (mAbs) followed by horseradish peroxidase-protein A and enhanced chemiluminescence detection.

The plasmid p2auxo.HIVA (Figure 2A)^26^ was transformed into the MTBVACΔ*lys* host strain to generate the recombinant MTBVAC.HIVA^2auxo^. The selection of positive recombinant MTBVAC.HIVA^2auxo^ colonies was performed by culturing the MTBVACΔ*lys* transformants on Middlebrook agar 7H10 medium without lysine supplementation. The MTBVAC.HIVA^2auxo^ strain harboring the lysine-complementing gene abolished the requirement for exogenous lysine, and colonies were observed in non-lysine-supplemented agar plates (Figure 2B).

The expression of the full-size chimeric 19-kDa signal sequence-HIVA protein (total weight, 64 kDa) was confirmed by western blot analysis of the MTBVAC.HIVA^2auxo^ cell lysates (Figure 2C). No HIVA protein expression was detected in recombinant MTBVAC strains harboring the p2auxo plasmid without heterologous insert (MTBVAC.∅^2auxo^, negative control).

This proper molecular characterization led us to prepare a master seed stock and derivative working vaccine stock for downstream experiments.

### *In Vitro* and *In Vivo* Maintenance of p2auxo.HIVA in MTBVAC.HIVA^2auxo^ Strain

**Figure 3:**
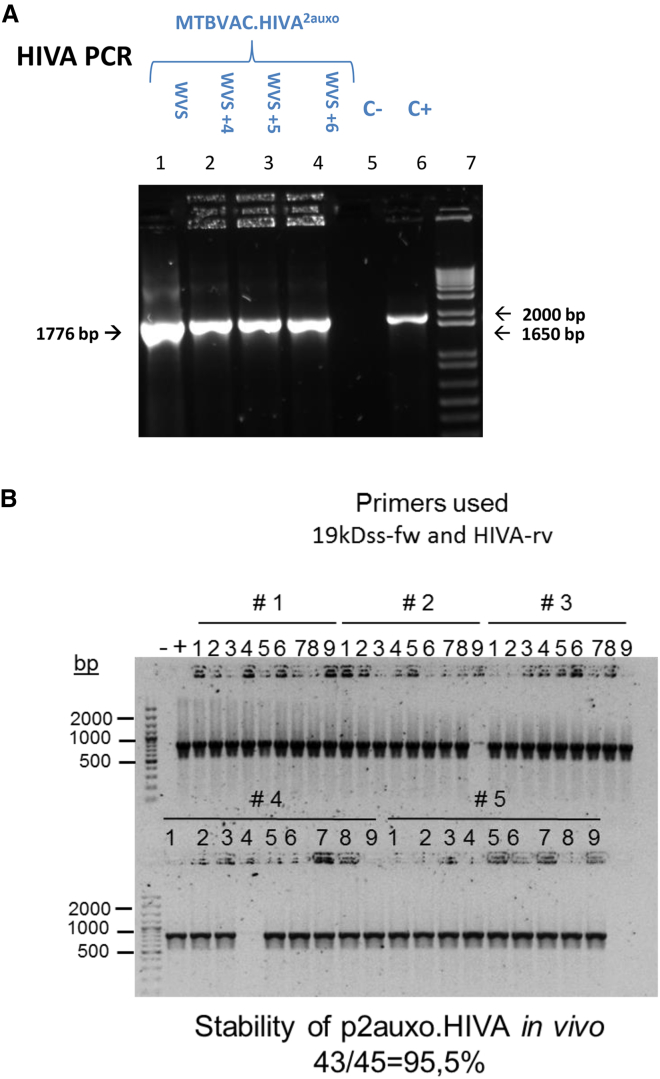
Genetic Stability of p2auxo.HIVA Plasmid DNA (A) *In vitro*. Serial passages of the working vaccine stock (WVS) were performed weekly (+1 to +6), and HIVA PCRs were used to check stability of the plasmid DNA. Lane 1, WVS MTBVAC.HIVA^2auxo^; lanes 2–4, passages +4, +5, and +6 WVS MTBVAC.HIVA^2auxo^; lane 5, H_2_0 (negative control); lane 6, positive control; lane 7, molecular weight marker. (B) *In vivo*. Spleens from SCID mice inoculated with 10^6^ CFU MTBVAC.HIVA^2auxo^ and used for safety experiments were harvested and plated on complete 7H10 supplemented with Lys and Km. The presence of p2auxo.HIVA plasmid in the colonies from these mice was analyzed by specific PCR using the pairs of primers to detect HIVA (19kDss-fw/HIVA-rv). Each number represents one colony and numbers with # symbol indicate colonies from the same animal. Minus and plus symbols indicate negative and positive controls of PCR, respectively. Plasmid maintained *in vivo* was calculated as the percent of positive colonies with respect to total colonies analyzed.

To assess the *in vitro* stability of the p2auxo.HIVA plasmid, subcultures of MTBVAC.HIVA^2auxo^ on selective media (no lysine supplementation) were carried out every 7 days. The maintenance of the p2auxo.HIVA plasmid DNA was evaluated by PCR analysis of HIVA and GlyA DNA-coding sequences. Bands corresponding to the HIVA DNA-coding sequence (Figure 3A) and to the *E. coli* GlyA-coding sequence (Figure S1A) were observed in all 6 MTBVAC.HIVA^2auxo^ subcultures (42 bacterial generations), indicating that there were no major genetic rearrangements in the *HIVA* and *glyA* genes of MTBVAC.HIVA^2auxo^ vaccine strain over the subsequent subculturing passages. *In vivo* stability of p2auxo.HIVA plasmid in MTBVAC.HIVA^2auxo^ was assessed in severe combined immunodeficiency (SCID) mice used in the safety trial. Homogenized spleens were plated on Lys-supplemented medium, and p2auxo.HIVA presence in the mycobacterial burden was analyzed by colony PCR using primers to detect the HIVA DNA-coding sequence (Figure 3B) and the *glyA* gene (Figure S1B). The analysis showed that 95.5% of the colonies retained the plasmid during *in vivo* infection.

### MTBVAC.HIVA^2auxo^ Prime and MVA.HIVA Boost Vaccination Schedule Elicited HIV-1- and PPD-Specific T Cell Responses in Mice

**Figure 4:**
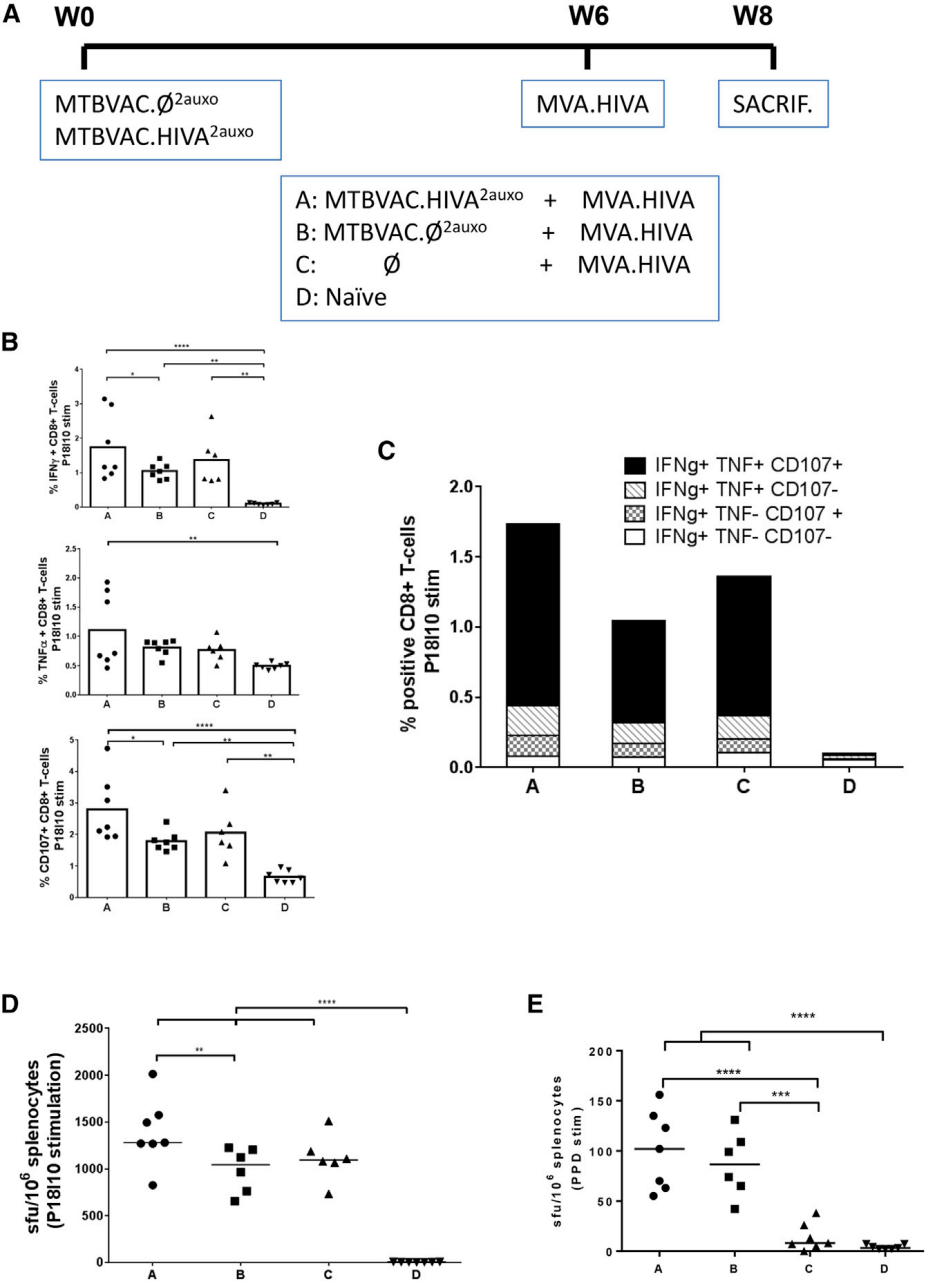
Induction of HIV-1- and *Mtb*-Specific T Cell Responses by the MTBVAC.HIVA^2auxo^ Prime MVA.HIVA Boost Regimen (A) Adult (7-week-old) mice were either left unimmunized or primed with 10^6^ CFU MTBVAC.HIVA^2auxo^ or MTBVAC.Ø^2auxo^ (intradermally) and boosted with 10^6^ plaque-forming units (PFUs) of MVA.HIVA (intramuscularly) 6 weeks post-MTBVAC inoculation. Mice were sacrificed 2 weeks later for T cell analysis. (B) Analysis of IFN-γ and CD107 vaccine elicited HIV-1-specific CD8+ T cell responses. The frequencies of cells producing cytokines are shown. Data are presented as means (SD; n = 7 for groups A, B, and D and n = 6 for group C). (C) The functionality of vaccine-induced CD8+ T cell responses was assessed in a multicolor intracellular cytokine staining assay. The group mean frequencies of single, double, and triple cytokine-producing P18-I10-specific cells are shown for the four vaccination groups. (D) Elicitation of specific T cell responses was assessed in an *ex vivo* IFN-γ enzyme-linked immunosorbent spot (ELISPOT) assay using the immunodominant P18-I10 CD8+ T cell epitope peptide. The median spot-forming units (SFUs) per 10^6^ splenocytes for each group of mice (n = 7 for groups A, B, and D and and n = 6 for group C) as well as individual animal responses are shown. (E) Purified protein derivative (PPD)-specific T cell responses elicited by MTBVAC.HIVA^2auxo^. Immune responses to mycobacteria were assessed in an *ex vivo* IFN-γ ELISPOT assay using PPD as the antigen. The median SFUs per 10^6^ splenocytes for each group of mice (n = 7 for groups A, B, and D and and n = 6 for group C) as well as individual animal responses are shown. Statistical analysis was performed by ANOVA plus Bonferroni multiple comparisons test (*p < 0.05, **p < 0.01, ***p < 0.001, and ****p < 0.0001).

We previously demonstrated that heterologous BCG.HIVA prime boosted with MVA.HIVA elicited high-quality HIV-1-specific T cell responses.^26^, ^29^, ^30^ In this study, we evaluated the specific HIV-1 T cell responses in adult BALB/c mice after intradermal immunization with MTBVAC.HIVA^2auxo^ or MTBVAC.Ø^2auxo^ prime and intramuscular MVA.HIVA boost (Figure 4A). The intradermal route mimics the administration performed in human BCG vaccination, and it has been shown to elicit a higher HIV-1-specific CD8+ T cell response in adult BALB/c mice.^31^ The immunogenicity readout was focused on the P18-I10 epitope, an immunodominant cytotoxic T-lymphocytes (CTL) epitope derived from HIV-1 Env and H-2D^d^ murine restricted, which was fused to HIVA immunogen to evaluate the immunogenicity in mice.

On day 0, adult mice were either left unimmunized or primed with MTBVAC.HIVA^2auxo^ or MTBVAC.Ø^2auxo^, and on week 6 the animals received an MVA.HIVA boost. Mice were sacrificed on week 8, and the functional quality of the elicited CD8+ T cells to produce interferon-γ (IFN-γ) and tumor necrosis factor alpha (TNF-α) and to degranulate (surface expression of CD107a) in response to P18-I10 peptide stimulation was measured by intracellular cytokine staining (ICS) (Figure 4B). We observed in adult mice that MTBVAC.HIVA^2auxo^ prime and MVA.HIVA boost induced higher frequencies of P18-I10 epitope-specific CD8+ splenocytes producing IFN-γ, TNF-α, and CD107 than mice primed with MTBVAC.Ø^2auxo^, MVA.HIVA alone, or naive mice. We found that MTBVAC.HIVA^2auxo^ prime and MVA.HIVA boost induced higher frequencies of trifunctional specific CD8+ T cells compared with the MTBVAC.Ø^2auxo^ priming and MVA.HIVA boost and with MVA.HIVA alone (Figure 4C).

The capacity of splenocytes from vaccinated mice to secrete IFN-γ was also assessed by the enzyme-linked immunosorbent spot (ELISPOT) assay. We observed the highest frequency of specific cells secreting IFN-γ when stimulated with P18-I10 in mice primed with MTBVAC.HIVA^2auxo^ and boosted with MVA.HIVA, 1,280 spot-forming units (SFU)/10^6^ splenocytes, compared to 1,043 SFU/10^6^ splenocytes obtained when mice were primed with MTBVAC.Ø^2auxo^ and 1,095 SFU/10^6^ splenocytes when mice were only boosted with MVA.HIVA (Figure 4D). The capacity of splenocytes from vaccinated mice to secrete IFN-γ after overnight stimulation with the *Mtb*-purified protein derivative (PPD) was also assessed by ELISPOT. The median SFUs per 10^6^ splenocytes were similar in mice primed with MTBVAC.HIVA^2auxo^ and MTBVAC.Ø^2auxo^ (102 and 86 SFU/10^6^ splenocytes, respectively; Figure 4E).

### MTBVAC.HIVA^2auxo^ Prime and MVA.HIVA Boost Were Well Tolerated in Mice

**Figure 5:**
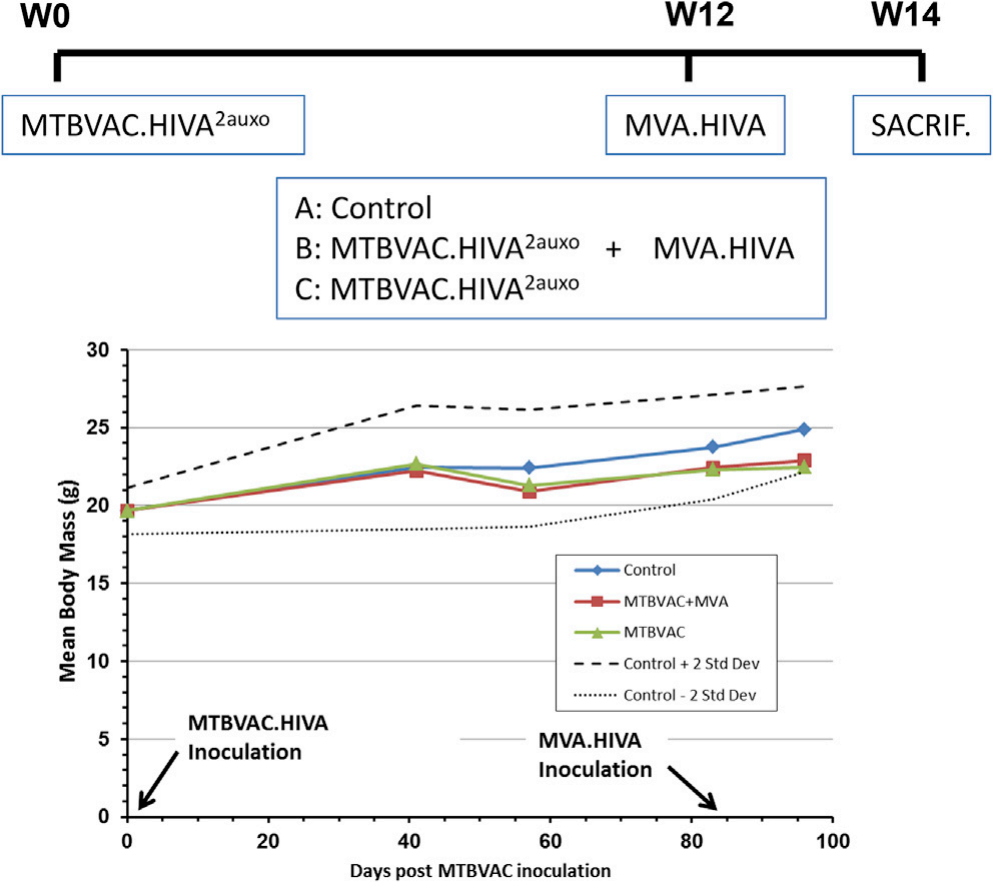
MTBVAC.HIVA^2auxo^ Prime and MVA.HIVA Boost Safety in Adult Mice Adult mice were either left unimmunized or immunized with 10^6^ CFU of MTBVAC.HIVA^2auxo^ by intradermal route and subsequently given a booster dose of 10^6^ PFU of MVA.HIVA at week 12 by intramuscular route. The body mass was recorded over time, and the mean for each group of mice is shown (n = 5). Data from control mice are presented as mean ± 2 SD (dashed lines). The weight differences between vaccinated and naive mouse group were analyzed at the final time point by ANOVA.

As shown in Figure 5, the body mass was monitored over time and recorded to depict any adverse events and body mass loss due to vaccination. To detect vaccine-derived adverse events, a 12-week period between MTBVAC.HIVA^2auxo^ and MVA.HIVA boost was established for this trial. Importantly, no statistically significant difference (by ANOVA) was observed between the vaccinated mouse groups and the control mouse group at the final time point. Furthermore, between weeks 1 and 14, the body mass monitored in all vaccinated mouse groups was found to lie between the mean ± 2 SD body mass curves in control mice. It is also important to mention that no mice died during the trial, and no local adverse events or associated systemic reactions were observed.

### MTBVAC.HIVA^2auxo^ Protective Efficacy against *M. tuberculosis* in Mice Was Similar to MTBVAC

**Figure 6:**
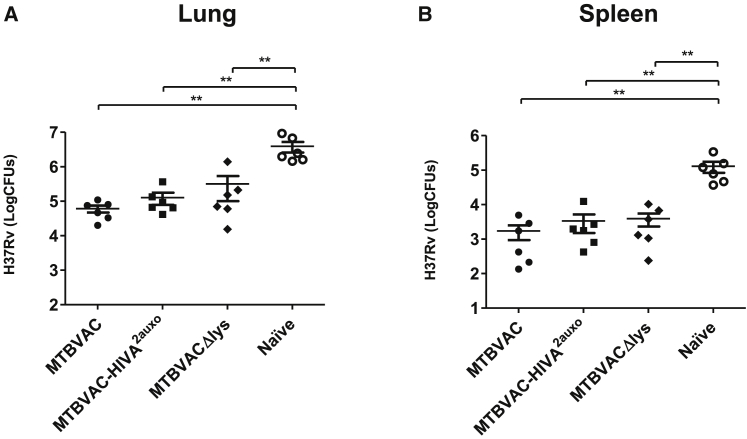
Efficacy of MTBVAC.HIVA^2auxo^ Vaccine against *M. tuberculosis* C57BL/6 mice were vaccinated subcutaneously with 10^6^ CFU of the strains indicated, MTBVAC, MTBVAC.HIVA^2auxo^, and MTBVACΔ*lys*, or naive (unvaccinated as control). At 8 weeks post-vaccination, mice were challenged by intranasal route with 200 CFU H37Rv. Bacterial burden was assessed in lungs (A) and in spleen (B) 4 weeks post-challenge. Data are expressed as mean ± SEM and compared by 2-way ANOVA test, using Bonferroni multiple comparison post-test (**p < 0.01).

We evaluated the efficacy of the bivalent vaccine strain MTBVAC.HIVA^2auxo^ with respect to the parental MTBVAC vaccine. Groups of 6 C57BL/6 mice were left unimmunized or vaccinated with MTBVAC, MTBVAC.HIVA^2auxo^, or MTBVAC*Δlys* by subcutaneous injection, a route previously used for efficacy studies in mouse models.^32^ At 7 weeks post-vaccination, the mice were challenged with the pathogenic H37Rv strain by the intranasal route. Bacterial load in lungs and spleens was examined 4 weeks post-challenge by plating homogenized organs on complete 7H10 medium (Figure 6). In all vaccinated groups, the bacterial reduction was significant with respect to the unvaccinated group, both in lungs and spleens. The auxotrophic strain MTBVAC*Δlys*, which was expected not to be able to survive without lysine, also displayed significant protection against *Mtb* H37Rv when compared to the naive group. No differences were found between the different MTBVAC strains tested, which validates the protective behavior of MTBVAC.HIVA^2auxo^ vaccine against *Mtb* despite the genetic manipulations introduced.

### MTBVAC.HIVA^2auxo^ Was Highly Attenuated in the SCID Mouse Model

**Figure 7:**
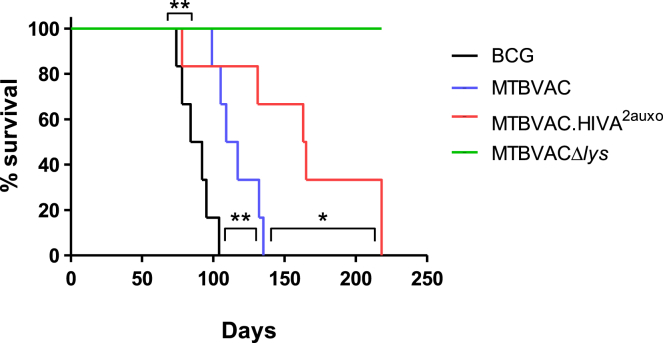
MTBVAC-HIVA^2auxo^ Safety in SCID Mice SCID mice were inoculated by intraperitoneal route with 10^6^ CFU BCG, MTBVAC, MTBVAC-HIVA^2auxo^, MTBVACΔ*lys*, or naive (unvaccinated as control). Analysis of survival was done applying the Mantel-Cox test (*p < 0.05 and **p < 0.01).

As well as affecting vaccine efficacy, genetic manipulation may also affect attenuation of live vaccines.^32^ With the aim of corroborating the attenuation status of MTBVAC.HIVA^2auxo^ and MTBVACΔ*lys* strains, SCID mice were inoculated with 10^6^ CFU by the intraperitoneal route, and the survival of animals was monitored (Figure 7). SCID mice are the reference model for safety assessments of live vaccines in preclinical TB studies, as recommended by regulatory bodies.^33^ Intraperitoneal, as well as intravenous, administration is a systemic inoculation route that allows rapid dissemination of the bacteria and, thereby, virulence assessments.^32^ The auxotrophic MTBVACΔ*lys* strain showed a hyper-attenuated profile; all mice inoculated with this strain survived until the endpoint of the experiment at week 31. Bacterial burden per spleen of these SCID mice vaccinated with MTBVACΔ*lys* at week 31 was approximately 5 × 10^3^ CFU (Figure S2), which demonstrated that this strain survived *in vivo*.

When we analyzed survival time of MTBVAC.HIVA^2auxo^-vaccinated mice, data revealed a marked attenuation profile (they survived approximately 160 days) when compared to the BCG-vaccinated mice (deceased by day 90) and the MTBVAC-vaccinated mice (deceased by day 120).

## Discussion

Despite the progress made in the development of a safe, effective, and affordable vaccine against HIV-1 and TB shortly after birth, the prevention of mother-to-child HIV-1 transmission via breast milk and childhood TB still remain great challenges.

The use of mycobacteria as a vaccine vector is an attractive option; on top of the previously mentioned advantages (cheap mass production, good safety profile, suitable for neonates, etc.), it induces a potent Th1 type immune response (the central defense mechanism against intracellular pathogens) in humans and mice.^34^, ^35^ Three experimental systems must be orchestrated to develop a recombinant *Mycobacterium*-based HIV vaccine: (1) a live vaccine vehicle based on *mycobacteria*, (2) an *E. coli*-mycobacterial expression vector without antibiotic resistance markers, and (3) an HIV immunogen design. In this study, we have engineered a novel live-attenuated vaccine for HIV-1 and *Mtb* infection that is vectored by a lysine auxotroph of MTBVAC,^16^ which expresses the HIV-1 clade A-derived immunogen HIVA.^25^

The use of mycobacterial vectors^20^, ^36^, ^37^ for antigen expression in *M. bovis* BCG,^20^, ^38^, ^39^ *M. smegmatis*,^40^ or *M. vaccae*^41^, ^42^ has been documented. Through a Barcelona-Oxford collaboration, we previously engineered a mycobacterial vaccine platform for HIV-TB by using lysine auxotrophic strains of BCG as vectors: an *E. coli*-mycobacterial expression vector containing an antibiotic-free selection system and different HIV immunogen designs to improve the specific HIV-1 immunogenicity.^26^, ^29^, ^31^, ^43^, ^44^

The auxotrophy-complementation strategy has also been used in the context of other intracellular pathogens as *Listeria monocytogenes* to express simian immunodeficiency virus (SIV) or HIV antigens.^45^ Despite being highly effective and safe in mice, including neonatal animals, the success observed in the murine model was not translatable upon immunogenicity and efficacy assessment of the *Lmdd-BdopSIVgag* in non-human primates.^46^ This should be taken into consideration during further stages of MTBVAC.HIVA^2auxo^ development.

In 2014, the promising live-attenuated *Mtb* vaccine, MTBVAC, was developed.^16^ It conferred improved immunogenicity and protection and had a higher safety profile compared to BCG in preclinical studies. Furthermore, MTBVAC has advanced through phase I (ClinicalTrials.gov: NCT02013245 and NCT02729571)^17^ and into phase II (ClinicalTrials.gov: NCT02933281 and NCT03536117) clinical trials.

It is important to recall that, of the 1,603 experimentally validated T cell epitopes present in *M. tuberculosis*, 433 of these epitopes were lost during the *in vitro* attenuation of BCG. Conversely, MTBVAC maintains the whole antigenic repertoire of the human pathogen *Mtb*.^47^ Further, MTBVAC displayed increased secretion and immunogenicity against some key antigens (such as those of the Ag85 complex) as a consequence of the *phoP* mutation.^48^, ^49^, ^50^ Thus, MTBVAC may well be a better platform than BCG when it comes to the expression of heterologous antigens, based on its higher immunogenicity.

The MTBVAC.HIVA^2auxo^ and MTBVAC.∅^2auxo^ strains constructed in this study grew properly without lysine supplementation in the medium, which demonstrated the usefulness of the auxotrophy-complementation system for the selection and maintenance of plasmids in *Mtb*-based vaccines.^26^, ^51^ Use of the lysine auxotroph-lysine complementation system was previously shown to increase plasmid stability and to prevent extensive genetic rearrangements in recombinant mycobacteria.^26^, ^43^, ^51^ Plasmid stability in recombinant mycobacteria has always been a critical and controversial issue. Méderlé et al.^52^ evaluated the genetic stability of recombinant BCG strains harboring episomal or integrative vectors expressing *nef* and *gag* genes from SIV. They observed a higher genetic stability *in vivo* and *in vitro* as well an increased duration of heterologous gene expression *in vivo* using the integrative plasmid.^52^ Recently, we published that the *in vitro* stability of the integrative plasmid p2auxo.HIVA^int^ was increased 4-fold compared with the BCG strain harboring the episomal plasmid.^30^ These results were in concordance with Méderlé et al. However, as there are more copies of episomal vector per cell, higher levels of recombinant protein can be expressed than from integrative vectors. Although our previous results and a number of others have demonstrated that integrative vectors are more stable than episomal vectors,^52^, ^53^ it has also been demonstrated that an initial, high level of antigen expression is necessary to prime an immune response.^52^ In this study, we demonstrated that the episomal p2auxo.HIVA plasmid transformed into the MTBVACΔ*lys* strain was retained *in vivo* over 220 days in SCID mice and after 42 bacterial generations *in vitro*. Sequencing of the recovered plasmids *in vitro* and *in vivo* would be desirable to accurately confirm the genetic stability of p2auxo.HIVA plasmid at the nucleotide level.

We assessed the HIV-1- and *Mtb*-specific T cell-mediated immune responses elicited after BALB/c prime-boost immunization with MTBVAC expressing HIVA immunogen (MTBVAC.HIVA^2auxo^). Mice were primed with MTBVAC.HIVA^2auxo^ or MTBVAC.∅^2auxo^ (plasmid with no HIVA DNA-coding sequence) and boosted with MVA.HIVA. This way, we were able to detect the non-specific immune responses due to intrinsic immunogenic properties of mycobacteria.^54^ The frequencies of CD8+ T cells producing IFN-γ, TNF-α, and CD107a were higher in mice vaccinated with MTBVAC.HIVA^2auxo^ in comparison with mice primed with MTBVAC.∅^2auxo^. A similar profile was observed in spleen cells producing IFN-γ after P18-I10 peptide stimulation measured by ELISPOT assay. These results are in concordance with our previously published data using BCG.HIVA^2auxo^ and BCG wild-type as priming agents.^26^ The extent of T cell polyfunctionality was correlated to protection against leishmaniasis in mice, HIV-1 in humans, and SIV in non-human primates.^55^ It has also been demonstrated that the magnitude and polyfunctionality of virus-specific CD8+ T cell responses were associated with the control of viral replication after SHIV-89.6P challenge in rhesus monkeys.^56^

The construction of *Mtb* auxotrophs (Δ*panCD*, *leuCD*, and *secA2*) expressing HIV or SIV antigens with the aim of developing a dual pediatric vaccine against TB and HIV has been described as oral vaccination of macaques at birth.^57^ However, unexpectedly, vaccinated infants required fewer SIV exposures to become infected compared to naive controls. Considering that the current TB vaccine, BCG, can induce potent innate immune responses and confer pathogen-unspecific trained immunity, they hypothesized that an imbalance between enhanced myeloid cell function and immune activation might have influenced the outcome of oral SIV challenge in A*Mtb*-SIV-vaccinated infants. Ideally, an appropriately targeted specific response directed toward beneficial epitopes of HIV would overcome the dangers of trained immunity while maintaining its positive aspects, which have been linked to decreased mortality in children receiving BCG, as well as lead to the control or prevention of HIV infection.^58^

When developing a dual vaccine against TB and HIV, it is important to confirm that the inclusion of HIV immunogens does not increase the metabolic burden in the TB vaccine that could affect its protective efficacy. We have already described this issue in previous publications, demonstrating that recombinant BCG-based HIV vaccine conferred the same level of protection as the wild-type.^29^ In the present study, we confirm that MTBVAC and MTBVAC.HIVA^2auxo^ confer equivalent levels of protection against *Mtb* challenge and, consequently, vaccine efficacy is not compromised after genetic manipulations for HIVA expression.

The recombinant MTBVAC.HIVA^2auxo^ strain showed an increased safety profile in comparison with the parental MTBVAC strain. This finding might support the potential use of this vaccine in individuals at risk of immunosuppression. On the other hand, the auxotrophic MTBVACΔ*lys* strain had a highly attenuated profile, with 100% survival in this group after 220 days of inoculation. This hyper-attenuated profile of MTBVACΔ*lys* was observed with other auxotrophic BCG and *Mtb* strains,^59^, ^60^, ^61^ due to lower viability and growth limitation without amino acid supplementation *in vivo*. Despite the growth limitation, the strain could be isolated in the spleens of SCID mice at the end of the trial, demonstrating that the auxotrophic strain was not fully cleared on day 220 after inoculation. It may suggest that the increased attenuation profile of the recombinant MTBVAC.HIVA^2auxo^ could be due to episomal plasmid loss. However, in our study, the persistence of plasmid was 95% in the isolated colonies from spleens of SCID mice inoculated with MTBVAC.HIVA^2auxo^.

We also demonstrated that MTBVAC.HIVA^2auxo^ prime and MVA.HIVA boost were well tolerated in adult BALB/c mice by monitoring and recording body mass over time. The findings were along the lines of previous observations with recombinant BCG expressing HIVA.^26^

In conclusion, the development of a recombinant MTBVAC-based HIV-TB vaccine may provide a new and improved tool for mycobacterial-based vaccine design. In the future, MTBVAC could be further developed as a vector to express optimized HIV immunogens and utilized in prime-boost vaccination protocols along with new boosting agents. It could, furthermore, be used as a novel mycobacterial vaccine platform for infectious diseases, such as malaria, whooping cough, and other tropical diseases, to prime protective immune responses shortly after birth.

## Materials and Methods

### Bacterial Strains and Culture Conditions

Mycobacterial cultures were grown in Middlebrook 7H9 broth medium or on Middlebrook agar 7H10 (Becton Dickinson) medium supplemented with albumin-dextrose-catalase (ADC; Difco Laboratories) and containing 0.05% Tween 80 and 20 μg/mL kanamycin. The L-lysine monohydrochloride (Sigma) was dissolved in distilled water and used at a concentration of 40 μg/mL. Mycobacterial suspensions for animal inoculation were prepared in PBS from frozen glycerol stocks previously titrated by plating serial dilutions.

### Construction of MTBVACΔ*lys*, MTBVAC.HIVA^2auxo^, and MTBVAC.∅^2auxo^ Strains

The strain MTBVACΔ*lys* was constructed following the bacterial artificial chromosome-recombineering (BAC-rec) strategy described by Aguilo et al.^27^ Briefly, the target gene *rv1293/lysA* was identified in the *E. coli* BAC library (kindly donated by Roland Brosch from Institut Pasteur, Paris). In this clone, the target gene *lysA* was interrupted with a kanamycin resistance cassette by heterologous recombination mediated by PCR product, which was amplified by using specific primers LysA-P1-pKD4-fw and LysA-P2-pKD4-rv (Table S1). The disrupted *lysA-Km* gene was amplified by PCR using ArgS1-fw and ThrB1-rv primers (Table S1). This PCR product containing the *lysA-Km* fragment was introduced into the MTBVAC genome by homologous recombination. MTBVACΔ*lys* recombinant colonies were selected by plating on 7H10-ADC supplemented with Km and Lysine, and proper recombination was checked by PCR using the pairs of primers Lys-fw/km-OUT1-rv and Lys-rv/km-OUT2-fw (Table S1).

For the construction of MTBVAC.HIVA^2auxo^ and MTBVAC.∅^2auxo^ strains, MTBVACΔ*lys* culture was grown until log phase and conditioned for electroporation by washing with 10% glycerol, according to the method described by Wards and Collins.^62^ Electrocompetent MTBVACΔ*lys* was transformed with 0.5–1 μg p2auxo.HIVA or p2auxo.∅,^26^ using a Bio-Rad gene pulser electroporator at 2.5 kV, 25 mF, and 1,000 Ω, and plated onto Middlebrook agar 7H10-ADC medium without lysine supplementation. Recombinant MTBVAC.HIVA^2auxo^ and MTBVAC.∅^2auxo^ colonies containing the corresponding plasmids p2auxo.HIVA and p2auxo.∅ were selected by PCR using the pair of primers 19kDss-fw/HIVA-rv and 19kDss-fw/Pglya-rv, respectively (Table S1). The MTBVAC.HIVA^2auxo^ colonies were assessed for heterologous HIVA protein expression, and clone 2 was selected and preserved using the seed-lot system. A master seed stock and derivative working stock, which we used also as a vaccine stock, were prepared and stored at −80°C with 20% glycerol as a preservative.

### SDS-PAGE and Western Blot Analysis

Cell lysates of mid-logarithmic-phase cultures of MTBVAC.HIVA^2auxo^ and MTBVAC.∅^2auxo^ strains were prepared, separated by SDS-PAGE using pre-cast 8%–16% gradient acrylamide gels (GeBaGel, Israel), and electroblotted onto polyvinylidene fluoride (PVDF) membranes using a semi-dry system (Bio-Rad). HIVA protein was detected using anti-Pk antibody (MCA 1360; Pierce, USA) with an enhanced chemiluminescence kit (WesternBright ECL; Advansta, USA). To visualize the bands, the LAS500 gel imaging system (GE Healthcare) was used.

### *In Vitro* Stability of the MTBVAC.HIVA^2auxo^ Strain

Six subcultures (∼42 bacterial generations) of MTBVAC.HIVA^2auxo^ (working vaccine stock), harboring the episomal p2auxo.HIVA plasmid DNA that contains the lysine-complementing gene, were grown in 7H9 broth on selective media (no lysine supplementation). Subcultures were performed every 7 days by transferring 100 μL stationary-phase culture to 5 mL fresh medium. PCR analysis of HIVA DNA-coding sequence and GlyA gene (Table S1) was performed to detect plasmid genetic rearrangements.

### Mouse Trials

For immunogenicity studies, adult (7-week-old) female BALB/c mice were left either unimmunized or immunized with MTBVAC.HIVA^2auxo^ or MTBVAC.Ø^2auxo^, and they were boosted with MVA.HIVA at doses, routes, and schedules outlined in the Figure 4 legend. On the day of sacrifice, individual spleens were collected, and splenocytes were isolated by homogenizing spleens using a cell strainer (Falcon; Becton Dickinson) and a 5-mL syringe rubber plunger. Following the removal of red blood cells with ACK lysing buffer (Lonza, Barcelona, Spain), the splenocytes were washed and resuspended in complete medium (R10 [RPMI 1640 supplemented with 10% fetal calf serum and penicillin-streptomycin], 20 mmol/L HEPES, and 15 mmol/L 2-mercaptoethanol).

Body mass was monitored over time and recorded to depict any adverse events and body mass loss due to vaccination. To detect vaccine-related adverse events, a 12-week period between MTBVAC.HIVA^2auxo^ prime and MVA.HIVA boost was established for this trial.

For protection studies, groups of 6 female C57BL/6 mice (Janvier Biolabs) were mock treated or subcutaneously vaccinated with 10^6^ CFU MTBVAC, MTBVACΔ*lys*, or MTBVAC.HIVA^2auxo^. At 8 weeks post-vaccination, mice were intranasally challenged with 200 CFU virulent *M. tuberculosis* H37Rv. Bacterial burden was assessed 4 weeks post-challenge by plating homogenized lungs and spleen on complete 7H10 medium.

For safety studies, groups of 6 female CB-17/Icr-Prkdc SCID mice (Janvier Biolabs) received a single intraperitoneal inoculation of 10^6^ CFU BCG Pasteur, MTBVAC, MTBVACΔ*lys*, or MTBVAC.HIVA^2auxo^. Mice were monitored for any sign of disease and body mass measurements were performed weekly. Experimental endpoint was set as a 20% body weight reduction. In the case of the MTBVACΔ*lys* group, the endpoint was established at 220 days post-inoculation, upon which surviving animals were humanely euthanized and bacterial load in spleen was quantified. Samples were also obtained from MTBVAC.HIVA^2auxo^ animals euthanized during the protocol to check for plasmid stability by colony PCR analysis using 19kDss-fw/HIVA-rv and HIVA-fw/Pglya-rv primers (Table S1).

### Peptides

For assessing the immunogenicity of HIVA in the BALB/c mice, the following peptides were used: H-2D^d^-restricted epitope P18-I10 (RGPGRAFVTI). The PPD (AJVaccines, Copenhagen, Denmark) was used to assess the immunogenicity induced by MTBVAC.

### *Ex Vivo* IFN-γ ELISPOT Assay

The ELISPOT assay was performed using a commercial IFN-γ ELISPOT kit (Mabtech, Nacka Strand, Sweden), following the manufacturer’s instructions. The ELISPOT plates (MSISP4510, 96-well plates with polyvinylidene difluoride membranes; Millipore, Billerica, MA) were coated with purified anti-mouse IFN-γ capture monoclonal antibody diluted in PBS to a final concentration of 5 μg/mL at 4°C overnight. A total of 5 × 10^5^ fresh splenocytes was added to each well and stimulated with 2 μg/mL P18-I10 peptide or 5 μg/mL PPD for 16 h at 37°C. Wells were washed four times with PBS 0.05% Tween 20 and twice with PBS before incubating with 100 μL 5-bromo-4-chloro-3-indoyl-phosphate/nitro blue tetrazolium substrate solution (Sigma). After 5–10 min, the plates were washed with tap water and dried, and the resulting spots were counted using an ELISPOT reader (Autoimmun Diagnostika, Strassberg, Germany).

### Intracellular Cytokine Staining

One million splenocytes were added to each well of a 96-well round-bottomed plate (Costar, Corning, NY), pulsed with 2 μg/mL P18-I10 peptide and kept at 37°C and 5% CO_2_ for 60 min, followed by the addition of GolgiStop (Becton Dickinson) containing monensin. After 5 h of incubation, the reaction was terminated by transferring the plate to 4°C. The cells were washed with wash buffer (PBS, 2% fetal calf serum, and 0.01% azide) and blocked with anti-CD16/32 (BD Biosciences) at 4°C for 30 min. All subsequent antibody stains were performed using the same conditions. Cells were then washed and stained with anti-CD8-PerCP (BD Biosciences) and anti-CD107a-fluorescein isothiocyanate (FITC), washed again, and permeabilized using the Cytofix/Cytoperm kit (BD Biosciences). Perm-wash buffer (BD Biosciences) was used to wash cells before staining with anti-IFN-γ-APC and anti-TNF-α-PE (BD Biosciences). Cells were fixed with CellFIX (Becton Dickinson) and stored at 4°C until analysis. All chromogen-labeled cells were analyzed in a Becton Dickinson FACScalibur, using the CellQuest software (Becton Dickinson) for acquisition and the FlowJo software (Tree Star, Ashland, OR) for analysis.

### Statistical Analysis

Immunogenicity data are shown as group means or group medians as well as individual responses. Statistical significance was determined by ANOVA and Bonferroni post-test. In safety experiments H37Rv bacterial burdens are shown as mean ± SEM. Groups were compared using one-way ANOVA and Bonferroni post-test. Survival data were analyzed applying Mantel-Cox test. In all cases, confidence intervals were as follows: *p < 0.05, **p < 0.01, and ***p < 0.01. GraphPad Prism 5.0 software was used for representation and statistical analysis of the data.

### Ethics Statement

All mice were kept under controlled conditions and observed for any sign of disease. The care and use of animals were performed accordingly with the Spanish Policy for Animal Protection RD53/2013, which meets the European Union Directive 2010/63 on the protection of animals used for experimental and other scientific purposes.

Immunological mouse experiments were approved by the local Research Ethics Committee (Procedure Med 365/16, Clinical Medicine, School of Medicine and University of Barcelona) and by the Ethical Committee for animal experimentation from the University of Barcelona, and they strictly conformed to the Generalitat de Catalunya animal welfare legislation. Experiments with SCID mice were carried out under Project License 17/17 and *M. tuberculosis* protection studies under Project License 50/14. Both procedures were approved by the Ethics Committee for Animal Experiments of the University of Zaragoza.

## Author Contributions

Conceptualization, J.G.-A., C.M., N.S., and J.J.-M.; Methodology, N.A., J.G.-A., N.S., and J.J.-M.; Investigation, E.B., S.U., and N.S.; Writing – Original Draft, E.B. and N.S.; Writing – Review & Editing, E.B., N.S., J.G.-A., C.M., and J.J.-M.; Funding Acquisition, C.M. and J.J.-M.; Supervision, E.B., S.U., N.A., and J.G.-A. The funders had no role in study design, data collection and analysis, decision to publish, or preparation of the manuscript.

## Conflicts of Interest

C.M. and J.G.-A. are co-inventors in patent applications entitled “TB vaccine,” filed by the University of Zaragoza (PCT/ES 2007/070051), and “Compositions for use as a prophylactic agent to those at risk of infection of TB, or as secondary agents for treating infected TB patients” (218382097.6-1112), University of Zaragoza/Biofabri. There are no other conflicts of interest. J.J.-M. and N.S. are co-inventors in a patent application entitled “Mycobacterium comprising expression vector with two auxotrophic selection markers and its use as vaccine” (EP 12382336.1), Laboratorios Esteve, S.A. and Fundación Privada Institut de Recerca de la SIDA. There are no other conflicts of interest. The rest of the authors certify that they do not have a commercial or other association that might pose a conflict of interest in the subject matter or materials discussed in this manuscript.

## Supplemental Information

Document S1. Figures S1 and S2 and Table S1

Document S2. Article plus Supplemental Information
