# Supplementary material for: MTBVAC-Based TB-HIV Vaccine Is Safe, Elicits HIV-T Cell Responses, and Protects against Mycobacterium tuberculosis in Mice
Source: Mol Ther Methods Clin Dev. 2019 Feb 7;13:253–64. doi: 10.1016/j.omtm.2019.01.014 (PMC6395831; doi:10.1016/j.omtm.2019.01.014)
Supplement: Document S1. Figures S1 and S2 and Table S1 [file mmc1.pdf]

OMTM, Volume *13*

Supplemental Information

MTBVAC-Based TB-HIV Vaccine Is Safe, Elicits HIV-T Cell Responses, and Protects against *Mycobacterium tuberculosis* in Mice

Esther Broset, Narcís Saubi, Núria Guitart, Nacho Aguilo, Santiago Uranga, Athina Kilpeläinen, Yoshiki Eto, Tomáš Hanke, Jesús Gonzalo-Asensio, Carlos Martín, and Joan Joseph-Munné

Supplementary Materials 1

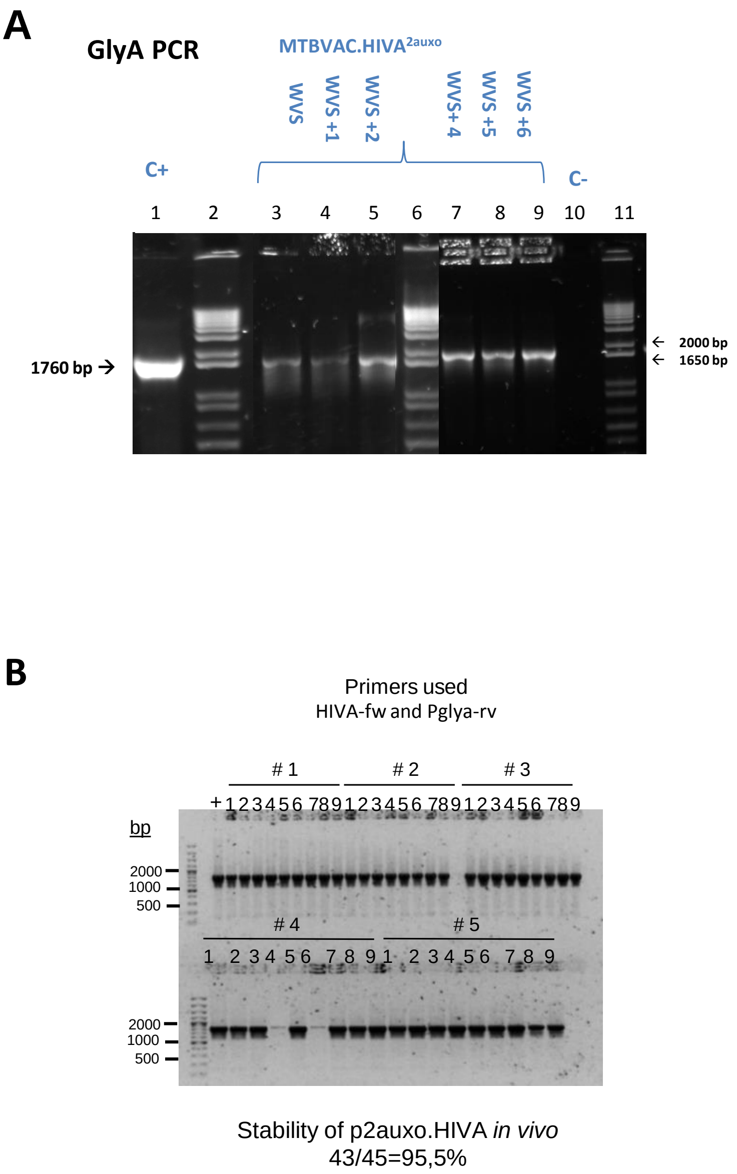

Supplementary Materials 1. Genetic Stability of p2auxo.HIVA plasmid DNA. (A) In vitro. Serial passages of the WVS were performed weekly (+1 to +6) and Gly A PCRs were used to check stability of the plasmid DNA. Lane 1: WVS MTBVAC.HIVA^{2auxo}; lanes 2, 3, 4: passages +4, +5 and +6 WVS MTBVAC.HIVA^{2auxo}; lane 5: $H_2$0 (negative control); lane 6: Positive Control ; lanes 7: Molecular weight marker. **(B) In vivo.** Spleens from SCID mice inoculated with 10^6 CFU of MTBVAC.HIVA^{2auxo} and used for safety experiments were harvested and plated on complete 7H10 supplemented with Lys and Km. The presence of p2auxo.HIVA plasmid in the colonies from these mice were analyzed by specific PCR using the pairs of primers to detect

GlyA (HIVA-fw/Pglya-rv). Each number represents one colony and numbers with # symbol indicate colonies from the same animal. Symbols – and + indicate negative and positive control of PCR respectively. Plasmid maintained *in vivo* was calculated as the percent of positive colonies respect to total colonies analyzed.

Supplementary Materials 2

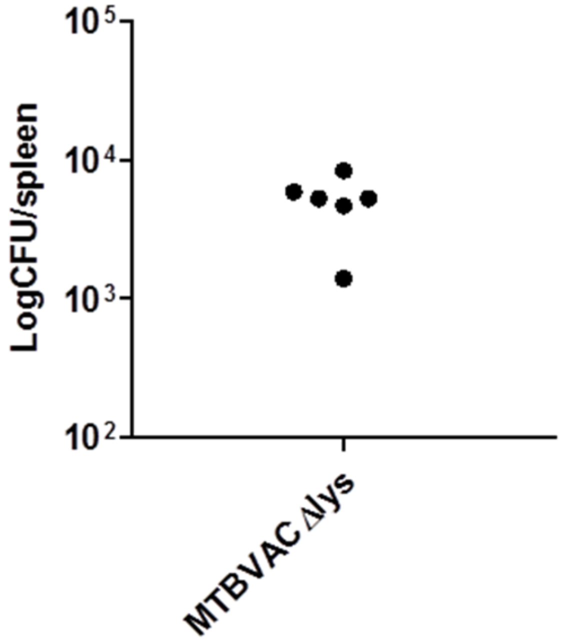

Supplementary Material 2: MTBVACΔ*lys* survival *in vivo* in SCID mice. Bacterial burden of SID mice was analyzed after 31 weeks of inoculation. Spleens were removed, homogenized and plated on complete 7H10 medium supplemented with Km and Lys.

Supplementary Table 1. Primer list

Primer	Primer sequence 5´->3´	Application
LysA-P1-pKD4- fw	CGAGGCGCACACCCACGAGTTCATCTCCACCGCGCACGAGGTGTAGGCTGGAGCTGCTTC	*Km* cassette cloning in *E. coli*-BAC128
LysA-P2-pKD4-rv	AAGCCGTCCACGTCGAAGATCTGCGAACCGATGTGGCTGCATATGAATATCCTCCTTAGT	
ArgS1-fw	GGCGATCGGCGTGGACGC	*lysA-Km* cassette cloning in MTBVAC
ThrB1-rv	CCCAGGATCGCCTTGCGCGAC	
19kDss-fw	ATATGAAGCGTGGACTGACG	p2auxo PCR check up
HIVA-rv	TCATCATCTCCTCCAGGGTG	
Pglya-rv	CGCACATTTCCCCGAAAAG	
HIVA-fw	GATCGAGGAGATCCAGAACAAG	
FW SpelSmal GlyA	TCACCCGGGACTAGTTGCTCATCCGGAGTGAAGAC	p2auxo plasmid integrity
RV SpelSmal GlyA	ACGCCCGGGACTAGTTCTAGAGGGCGGATTTGTCCTAC	
